# N-Induced Species Loss Dampened by Clipping Mainly Through Suppressing Dominant Species in an Alpine Meadow

**DOI:** 10.3389/fpls.2022.815011

**Published:** 2022-03-22

**Authors:** Wenyuan Wu, Xiangtai Wang, Zhengwei Ren, Xianhui Zhou, Guozhen Du

**Affiliations:** State Key Laboratory of Grassland and Agro-Ecosystems, School of Life Sciences, Lanzhou University, Lanzhou, China

**Keywords:** competitive ability, niche differentiation, functional traits, CWM, CV_NND, grassland

## Abstract

Nitrogen addition and clipping can exert substantial impact on species diversity but their interactions and the underlying mechanisms still remain unclear. Resource competition theory holds that sufficiently strong competitive ability of dominant species can lead to the losses of subordinate species through competitive exclusion, while niche differentiation theory suggests that the persistence of subordinate species in competitive systems can be promoted by guaranteeing positive growth rates of rare species. Taking advantage of a field experiment with nitrogen addition (10 g N m^–2^ year^–1^) and different clipping intensities (2, 15, and 30 cm) treatments in a Tibetan alpine meadow across 2015–2020, we assessed the relative importance of competitively dominant species and niche differentiation in driving species diversity changes *via* using community weighted mean (CWM) and variation coefficient of nearest neighbor distance (CV_NND) of functional traits including height, specific leaf area (SLA) and leaf dry matter content (LDMC). We show that nitrogen enrichment drove a strong plant diversity loss (*P* < 0.001). Clipping at different intensities had little effect on species diversity, but it can reduce the N-induced diversity loss. Nitrogen addition and clipping caused changes in community diversity were mainly indirectly attributed to their effects on community functional composition, and the competitive ability of dominant species. Nitrogen increased the CWM of functional traits to improve the competitive ability of dominant species. In contrast, clipping influenced species diversity positively by decreasing CWM_height_ (*P* < 0.001), and also negatively by increasing CWM_SLA_ (*P* < 0.001) and decreasing CV_NND_SLA_ (*P* < 0.05). Interacting with N addition, clipping resulted in a neutral effect on species diversity, because clipping could offset the negative effects of nitrogen addition through an opposite effect on CWM_height_. This study provides new insights into the mechanisms of diversity maintenance with respect to nitrogen addition and clipping. Thus, clipping is recommended as a useful management strategy to alleviate the species loss caused by nutrients enrichment and maintain the diversity of grassland ecosystems.

## Introduction

Increasing human activities and environmental changes have caused the loss of species in many ecosystems, especially in grasslands (Borer et al., 2014; DeMalach et al., 2017; Harvey et al., 2018; Brandt et al., 2019; Molina et al., 2021). The dramatic decline in grassland diversity is often attributed to increased nutrient enrichment (Stevens et al., 2004; Harpole et al., 2016, 2017; Seabloom et al., 2021) or changed land-use (Golodets et al., 2011; Herrero-Jáuregui and Oesterheld, 2017; Zhang et al., 2018; Rahmanian et al., 2020). One of the potential mechanisms explaining species coexistence is the competitive ability of dominant species (Mortensen et al., 2017; Saiz et al., 2019). Sufficient functional dominance can promote competitive exclusion, leading to the losses of subordinate species (Wilsey and Polley, 2004; Hautier et al., 2009). Niche differentiation is the other possible mechanism maintaining species coexistence (Harpole and Tilman, 2007; Isbell et al., 2009; Doležal et al., 2018), which has been shown to promote the persistence of subordinate species in communities (Palmer, 1994; Gonzalez and Loreau, 2009). However, the relative importance of competitively dominant species and niche differentiation remains unclear.

Nutrient addition is a commonly used management practice to improve grassland production (Schellberg et al., 1999; Conant et al., 2001; Socher et al., 2012). While increasing community productivity, nutrient enrichment is also a major driver of biodiversity loss (Phoenix et al., 2006; Southon et al., 2013; Borer et al., 2014). Since plants have different resources acquisition ability, nutrient enrichment may cause asymmetric resource availability and further result in asymmetric competition amongst competing plants (Rajaniemi, 2002; Niu et al., 2008; Hautier et al., 2009; Goodwillie et al., 2020). For example, Rajaniemi (2002) attributed the loss of diversity to increased underground competition after N-P-K fertilization. Similarly, Hautier et al. (2009) showed that nitrogen addition can reduce plant diversity *via* inducing aboveground light competition. Moreover, nutrient enrichment can alter the competitive intensity *via* shifting plants’ resources allocation strategies. For example, Niu et al. (2008) found that fertilization significantly decreased leaf allocation for forbs but increased leaf allocation for grasses, thereby resulting in an increase in competitive ability of grasses. Due to the increased light limitation, nitrogen addition may also favor taller species thereby excluding almost all shorter species.

Land-use management, such as grazing or clipping also plays an important role in maintaining plant diversity in grasslands (Klimek et al., 2008; Speed et al., 2013; Beck et al., 2015; Bakker et al., 2016; Doležal et al., 2018; Kapás et al., 2020). Since high biodiversity can be maintained under moderate grazing intensity (Grime, 1973; Connell, 1978; Li et al., 2021), regular clipping (simulated grazing) also is likely to produce similar effects (Antonsen and Olsson, 2005; White et al., 2014; Nagata et al., 2016; Smith et al., 2018; Yang et al., 2019). For instance, clipping at a low intensity usually imposes moderate disturbance which can significantly raise plant diversity in grasslands, whereas high levels of clipping lead to a decline in species diversity (Socher et al., 2012). *Via* inhibiting the competitive ability of dominant species, clipping was able to decrease the advantage of dominant species and release forbs species that can withstand grazing pressure (such as low-stature and creeping-growth forms) from the competition, thereby contributing to the maintenance of diversity (Isbell et al., 2009; Doležal et al., 2018). Moreover, grazing or clipping may induce niche differentiation of species in plant communities, which is a critical mechanism to promote species co-existence (Pierce et al., 2007; Niu et al., 2015; Wang et al., 2021). For example, Niu et al. (2015) found that grazing promoted species diversity *via* inducing differentiation of leaf phosphorus in an alpine meadow; Wang et al. (2021) also found that clipping promoted the asynchronous response of subordinate species (Doudová and Douda, 2020), and ultimately increased community species diversity. Thus, grazing or clipping may alleviate the negative effects of fertilization on plant community diversity.

To distinguish the relative importance of competitively dominant species and niche differentiation for diversity changes in the context of clipping and nitrogen addition, we used a trait-based approach. Since community weighted means (CWM) were weighted by the relative contribution of species to calculate the mean trait value for a given community (Díaz et al., 2007), the metric can capture the effect of dominant species (Avolio et al., 2019) and represent the competitive ability of dominant species. To indicate niche differentiation effects, the variation coefficient of nearest neighbor distance (CV_NND) of traits was used (Schöb et al., 2012). CV_NND was calculated using the relative distances between traits (Jung et al., 2010) which can quantify trait spacing and indicate the microscale environmental heterogeneity and resource allocation (Schöb et al., 2012).

Here, we conducted a 5-year experiment with nitrogen addition and clipping in an alpine meadow on the eastern Qinghai-Tibet Plateau to assess the independent and interactive effects of nitrogen addition and clipping on plant community diversity. We measured three plant functional traits [i.e., height, specific leaf area (SLA), and leaf dry matter content (LDMC)] for most species (coverage greater than 5%) and calculated indices relating to the competitive ability of dominant species and niche differentiation at the community level (i.e., CWM and CV_NND of each trait, respectively). These analyses allow us to examine the relative importance of the two ecological mechanisms in driving plant diversity. We hypothesized that: (1) the negative effect of nitrogen addition on the diversity of plant communities would be counteracted by clipping; (2) the decline in species diversity due to nitrogen addition is often accompanied by a dramatic increase in the height and competitiveness of dominant species; however, (3) clipping may increase plant diversity or alleviates negative effects of nitrogen addition on plant diversity *via* reducing the competitive ability of dominant species and increasing niche differentiation.

## Materials and Methods

### Study Site and Experimental Design

Our field experiment was carried out at the Research Station of Alpine Meadow and Wetland Ecosystems of Lanzhou University (Maqu Branch Station), Gansu province, northwestern China, at the elevation of approximately 3,500 m (33° 40′N, 101° 52′E). According to the past 35-year observation (from the Maqu County Meteorological Bureau), the mean annual precipitation in the region is about 620 mm, and mainly distributed in the short summer. The average annual temperature is 1.2^°^C, ranging from −10^°^C in January to 11.7^°^C in July. The vegetation is characterized by typical alpine meadow, and the soil is subalpine meadow soil. The natural habitat is dominated by perennial sedges (e.g., *Kobresia graminifolia*), Gramineae (e.g., *Elymus nutans* and *Poa poophagorum*), and forbs (e.g., *Anemone rivularis*). The study area had received no fertilizer or clipping before this experiment. During the study, fencing was used to prevent large mammal grazing.

**FIGURE 1:**
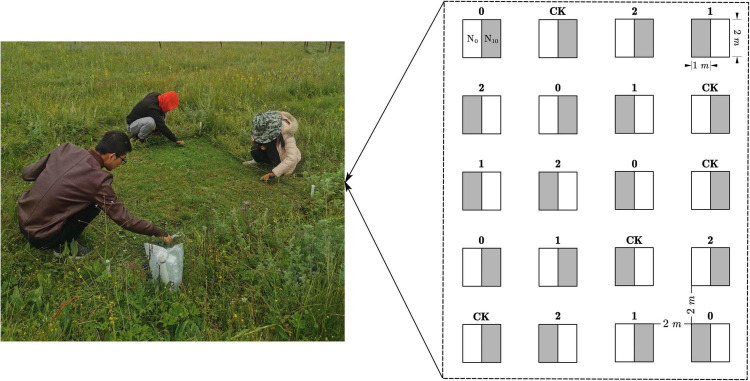
Schematic representation of the experiment design. Twenty 2 × 2 m plots were laid out in four columns and five rows with a 2 m-wide buffer zone between the plots. Labels above the boxes represent the clipping treatments that were randomly assigned to the plots (CK: no clipping; 0: high intensity; 1: moderate intensity; 2: low intensity). Each 2 **×** 2 m plot was divided into two subplots, one was dedicated to the ambient N (N_0_, subplot A white), another to nitrogen addition treatment (N_10_, subplot B gray).

Our experiment was established at a flat site in May 2014. Within the experimental area, twenty 2 × 2 m plots were laid out in four columns and five rows with a 2 m-wide buffer zone between the plots (Figure 1). Four clipping treatments and five replicates were randomly assigned to plots and applied every week from June to July every year. By removing plant above ground parts at different heights, three clipping intensity levels were implemented including (1) low intensity (stubble height: 30 cm), (2) moderate intensity (stubble height: 15 cm), and (3) high intensity (stubble height: 2 cm). Each plot was divided into two subplots, resulting in a total of 40 subplots. Since the threshold for changes in biomass, species diversity and community composition in response to nitrogen addition in mature Eurasian grasslands is about 10.5 g N m^–2^ year^–1^ (Bai et al., 2010), we randomly assigned each subplot to one of two N treatments: ambient (N_0_, control) or N addition (N_10_, 10 g N m^–2^ year^–1^, in the form of NH_4_NO_3_). Fertilization was carried out once a year in late May every year.

### Vegetation Sampling and Plant Functional Trait Measurements

Species richness and species abundance were recorded annually within a 0.5 × 0.5 m quadrat that was randomly placed within each subplot in late-August from 2015 to 2020. In May 2018, we measured three plant functional traits for each species, including plant height (H, cm), (SLA, cm^2^/g) and LDMC, which are easy to measure and closely related to resource acquisition and utilization. Plant height has a direct impact on plant competition, because taller species can gain a competitive advantage through preferential exposure to light (Westoby et al., 2002). SLA is positively related to photosynthetic capacity, leaf longevity, relative growth rate, and competitive ability, therefore increased CWM_SLA_ represents a good indicator of eutrophication (Ordoñez et al., 2009). LDMC is also widely used as an indicator of plant resource-use strategy (Vaieretti et al., 2007).

In each subplot, 10 individuals of each species were randomly selected and their heights were recorded. If the number of individuals was less than 10, all individuals of the species were measured. One mature and complete leaf was selected from each individual per species in all subplots, and the fresh weight of each leaf was weighed with an electronic analytical balance. Leaf areas were calculated with ImageJ software after scanning by an Epson-V300 scanner. After dried at 75^°^C for 48 h to a constant weight, the biomass of each leaf was weighed. Then the SLA was calculated as the ratio of leaf dry weight to leaf area, and LDMC as the ratio of leaf dry mass to leaf fresh weight.

### Indices of Plant Functional Diversity

Two functional metrics, CWM and coefficient of variation of nearest neighbor distance (CV_NND), were calculated.

The CWM value of each trait was calculated in each community according to Lavorel et al. (2008):

$$C\,W\,M=\sum_{i=1}^{n}p_{i}\times t\,r\,a\,i\,t_{i}$$

where p_i_ and trait_i_ are respectively the relative abundance and trait value of species i in the community. CWM is strongly driven by the trait values of dominant species; a high CWM value indicates a strong role of dominant species in the community.

We also estimated individual trait differentiation by calculating the CV_NND of each trait individually. According to Schöb et al. (2012), the CV_NND values were calculated as the coefficient of variation of differences between successive trait values of neighbors within a plot. With CV_NND, a lower value reflects niche differentiation (i.e., even spacing of traits) (Jung et al., 2010), while a higher value indicates clumping of species in trait space (Schöb et al., 2012).

### Statistical Analyses

To meet the assumptions of normal distribution and variance homogeneity, the data were log-transformed when necessary. Two-way ANOVAs were employed to assess the effects of clipping, nitrogen addition, and their interaction, on species diversity and CWM and CV_NND of different functional traits. Two-way ANOVAs with Tukey tests were used to test for differences in each index among treatments.

Structural equation modeling (SEM) was used to explore effects of clipping, nitrogen addition, and their interaction on Shannon diversity through CWM and CV_NND of each trait. Our experimental treatments (nitrogen addition and clipping) could impose certain environmental constraints within the community that limited the range of trait values (Díaz et al., 1998), which can be reflected in functional composition and functional dispersion (denoted by CWM and CV_NND, respectively). Therefore, we conducted SEM according to *a priori* model with the following premises: (1) clipping, nitrogen addition and their interaction could directly affect CWM and CV_NND of different functional traits; (2) Shannon diversity was indirectly mediated by clipping, nitrogen addition and their interaction through CWM and CV_NND of different traits.

To test the goodness of SEMs, we used a combination of χ^2^ test, root mean square error of approximation (RMSEA) test and comparative fit index (CFI). A non-significant χ^2^ and RMSEA test, and CFI > 0.9 indicate a good fit of the model to the data.

All data were analyzed using R software, version 4.1.0 (R Development Core Team, 2021). Shannon diversity was calculated with the “vegan” package (Oksanen et al., 2020), while the SEMs were conducted using the “lavaan” package (Rosseel, 2012).

## Results

**FIGURE 2:**
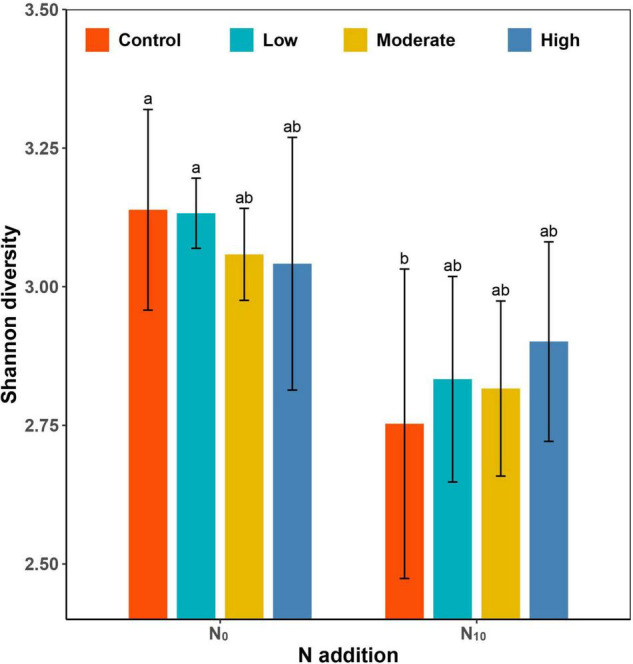
Barplot showing the differences in Shannon diversity among fertilization (N_0_: control, N_10_: nitrogen addition) and clipping (i.e., control, low intensity, moderate intensity, high intensity) treatments. Values (±SE) are means of five replicates of each treatment. Different letters denote significant differences between treatments (*P* < 0.05, Tukey’s HSD tests).

Species richness (*P* < 0.001) and Shannon diversity (*P* < 0.001) were significantly lower in the N_10_ plots than in N_0_ plots. Clipping at different intensities did not cause significant changes in Shannon diversity compared with the control (Table 1 and Figure 2). However, clipping alleviated the decline of plant diversity under fertilized plots. Since the changes of species richness in each treatment were consistent with Shannon diversity, only Shannon diversity was used to illustrate the results in this paper.

**TABLE 1:** *P*-values of two-way ANOVA to evaluate the effect of clipping, nitrogen addition and their interaction on community indices.

Community indices	Clipping	Nitrogen	Clipping × N
Richness	*0*.*085*	** < 0.001**	0.304
Shannon diversity	0.952	** < 0.001**	0.485
CWM_height_	** < 0.001**	** < 0.001**	** < 0.001**
CWM_SLA_	** < 0.001**	** < 0.001**	** < 0.01**
CWM_LDMC_	** < 0.001**	0.138	** < 0.001**
CV_NND_height_	** < 0.001**	0.118	** < 0.05**
CV_NND_SLA_	** < 0.05**	0.317	0.225
CV_NND_LDMC_	** < 0.001**	0.764	0.264

*Probabilities considered statistically significant (P < 0.05) and marginally significant (P < 0.1) are indicated in bold and italic typeface, respectively. CWM, community weighted mean; CV_NND, coefficient of variation of nearest neighbor distance; SLA, specific leaf area; LDMC, leaf dry matter content.*

**FIGURE 3:**
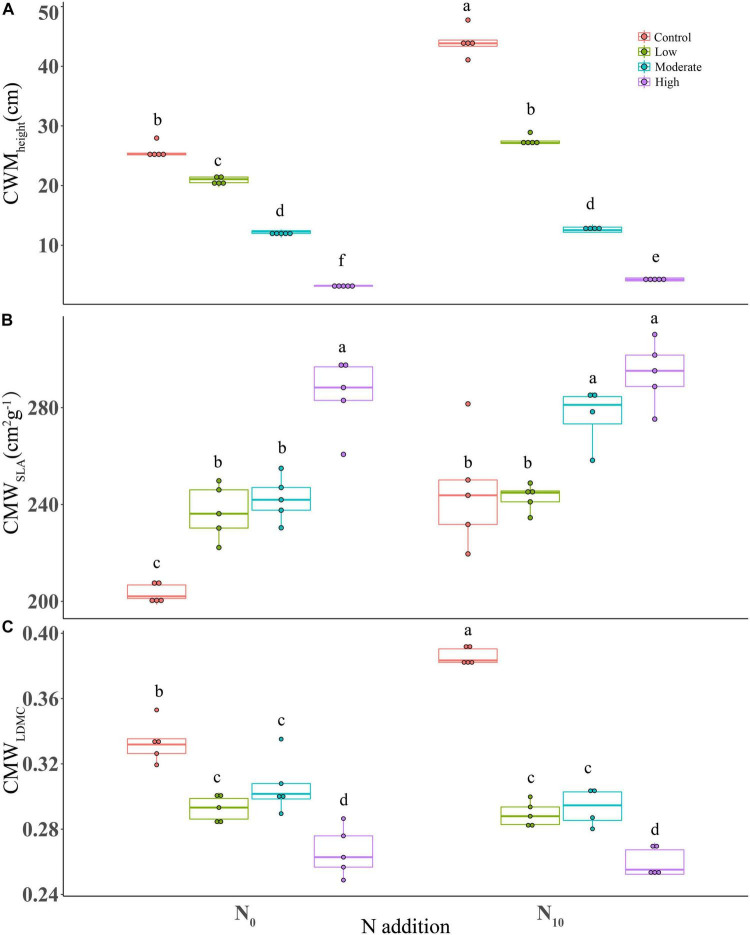
Boxplots showing the differences in CWM of functional traits among fertilization (N_0_: control, N_10_: nitrogen addition) and clipping (i.e., control, low intensity, moderate intensity, high intensity) treatments. **(A)** height, **(B)** specific leaf area, and **(C)** leaf dry matter content. CWM, community weighted mean. The box signifies the upper and lower quartiles, and the whiskers extend up to 1.5 times that intra-quartile range. Median is represented by horizontal line. For a given trait, different letters between treatments donate significant differences (*P* < 0.05, Tukey’s HSD tests).

Nitrogen addition, clipping and their interaction had significant effects on the community weighed means for the three functional traits (Table 1). Specifically, nitrogen application significantly increased CWM for plant height and SLA (height, *P* < 0.001; SLA, *P* < 0.001; LDMC, *P* = 0.138) (Table 1), indicating a positive effect on the competitive ability of dominant species. Clipping significantly increased CWM_SLA_ (*P* < 0.001), but decreased CWM_height_ (*P* < 0.001) and CWM_LDMC_ (*P* < 0.001) (Figure 3). Increasing clipping intensities could partially offset the changes of CWM traits caused by N addition. At high and moderate intensities, clipping counteracted the differences in CWM_height_ due to nitrogen addition (Figure 3A). The increase in CWM_SLA_ under nitrogen addition could be offset by clipping at low and high intensities (Figure 3B). Similarly, N-induced increase in CWM_LDMC_ could also be alleviated under different intensities of clipping (Figure 3C).

**FIGURE 4:**
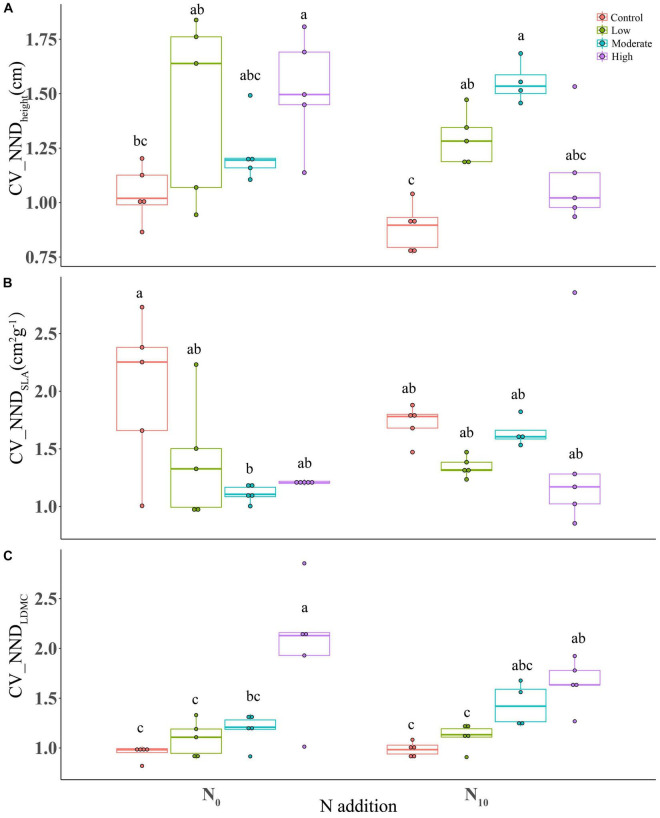
Boxplots showing the differences in CV_NND of functional traits among fertilization (N_0_: control, N_10_: nitrogen addition) and clipping (i.e., control, low intensity, moderate intensity, high intensity) treatments. **(A)** height, **(B)** specific leaf area, and **(C)** leaf dry matter content. CV_NND, coefficient of variation of nearest neighbor distance. The box signifies the upper and lower quartiles, and the whiskers extend up to 1.5 times that intra-quartile range. Median is represented by horizontal line. For a given trait, different letters between treatments donate significant differences (*P* < 0.05, Tukey’s HSD tests).

Nitrogen addition had no significant effects on the coefficient of variation of nearest neighbor distance (CV_NND) of all traits (height, *P* = 0.118; SLA, *P* = 0.317; LDMC, *P* = 0.764) (Table 1). Clipping at high intensity remarkably increased the CV_NND of plant height and LDMC (Figures 4A,C), but significantly decreased CV_NND_SLA_ at moderate intensity (Figure 4B). The interaction between clipping and N addition had a significant negative effect on CV_NND_height_ (Table 1). High clipping intensity significantly reduced the variation of CV_NND_height_ under N addition (Figure 4A).

**FIGURE 5:**
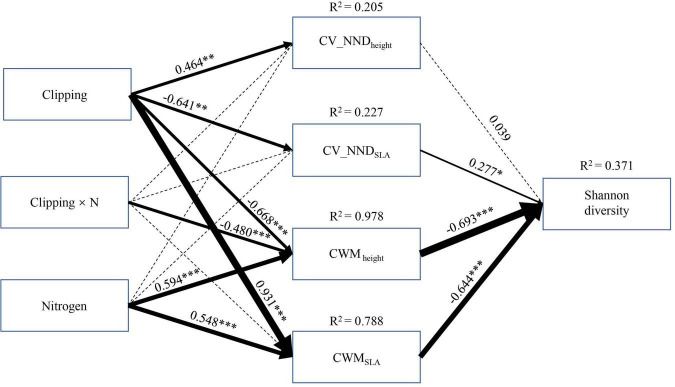
Structural equation modeling of the clipping by nitrogen addition effects on the Shannon diversity of plant community. For abbreviations see Figures 3, 4. df = 19, *P*(Chi-square) = 0.078, CFI = 0.980, *P*(RMSEA) = 0.141. Black solid arrows indicate a significant effect (at the level *P* < 0.05), and black dashed arrows indicate non-significant effect (at the level *P* > 0.05). Values associated with solid arrows represent standardized path coefficients, which are also indicated by arrow width. R^2^ values associated with response variables indicate the proportion of explained variation by relationship with other variables. Black solid arrows indicate a significant effect (at the level *P < 0.05, ^**^P < 0.01, ^***^P < 0.001).

The SEM model showed that the CWM of functional traits was the most important attribute in influencing community diversity when including both direct and indirect effects (Figure 5). Community diversity was mainly altered by treatments and their interaction indirectly rather than their direct effects (Figure 5). Specifically, nitrogen addition showed a negative effect on Shannon diversity indirectly through increasing CWM_height_ and CWM_SLA_, whereas the combination of N addition and clipping positively affected Shannon diversity mainly through decreasing CWM_height_. Clipping indirectly affected Shannon diversity simultaneously through CWM and CV_NND of different functional traits. In particular, clipping had a positive effect on Shannon diversity by decreasing CWM_height_ and a negative effect on diversity by increasing CWM_SLA_. At the same time, clipping could negatively affect Shannon diversity through decreasing CV_NND_SLA_, but the effect of this pathway was weak.

## Discussion

Nitrogen enrichment drove a strong plant diversity loss through the increasing competitive ability of dominant species. Clipping could affect both the competitiveness of dominant species and niche differentiation. In particular, clipping reduced the N-induced diversity loss mainly by suppressing competitive dominant species. We also observed significant interaction between nitrogen addition and clipping on dominant species’ height, a trait that closely associated with light competition. Together, the results show that competitive ability of dominant species plays a substantial role in diversity maintenance under nitrogen addition and clipping conditions.

Extensive studies have shown that enhanced competition for light is a major mechanism for the diversity loss under fertilization (Hautier et al., 2009; Socher et al., 2012; Yang et al., 2012; Goodwillie et al., 2020). According to the light competition hypothesis (Hautier et al., 2009; Borer et al., 2014; DeMalach et al., 2017), N enrichment releases plant species from symmetrical competition for belowground resources, intensifying asymmetric competition for aboveground light. Taller species generally capture more light resources than shorter ones, which could further expand their competitive advantages, ultimately resulting in the competitive exclusion (Goodwillie et al., 2020). In our study, N-induced negative effects on Shannon diversity were mainly due to the increasing CWM of plant height and SLA, so we inferred that nitrogen addition may decrease species diversity by favoring taller dominant species. Increasing CWM_height_ and CWM_SLA_ under nitrogen addition are expected to increase canopy height and reduce light available for smaller species in the understory, resulting in a decline in species diversity (Hautier et al., 2009). Since rare species are generally of smaller stature and occupy higher diversity (Lavergne et al., 2004; Pfestorf et al., 2013), reduced understory light due to N addition may reduce the number of rare species. Thus, the negative effects nitrogen addition imposed on Shannon diversity were mainly through promoting the competitive advantage of dominant species.

Most previous studies have shown that clipping can increase plant diversity in diverse ways, such as increasing light availability (Borer et al., 2014), reducing species dominance (Lepš and Wan, 2014), promoting rare species regeneration (Wilsey and Martin, 2015) and enhancing seedling germination (Foster and Gross, 1998). A few studies have found that clipping has a weak or even negative effect on plant diversity. For instance, Socher et al. (2012) reported that clipping early in the growing season and clipping at high frequency both decreased biodiversity. Morgan (2015) also found a decrease in diversity when clipping produced excessive litters which suppressed seedling establishment. However, here we found that clipping had little effect on species diversity. With the increasing intensities, clipping promoted dominant species by increasing CWM_SLA_, and simultaneously exerted suppressive effects by decreasing CWM_height_, indicating that broadleaf forbs with lower height and higher SLA such as *A. rivularis* were favored under clipping treatments. While clipping could help maintain species diversity by suppressing dominant resource competitors such as tall grasses (Borer et al., 2014), broadleaf species with higher SLA could also reduce light availability to understory and outcompete species that are less effective at light capture (Yang et al., 2015). Besides, clipping could also promote niche differentiation through the decreasing CV_NND_SLA_ under moderate intensity. There may be trade-off between these pathways, resulting in no significant clipping effect on diversity in our study.

Our results showed that clipping could alleviate the negative effect of nitrogen enrichment on species diversity to some extent. Under fertilized plots where nutrients reinforce the competitive advantage of dominant species (Farrer and Suding, 2016), clipping could inhibit dominant taller species through the decreasing CWM_height_, which alleviates light competition and promotes random colonization of rare local species (Song et al., 2020), thus maintaining local-scale plant diversity (Hillebrand et al., 2007). We also found a significant negative effect of the interaction between nitrogen addition and clipping on CWM_height_. However, the effects of nitrogen addition and clipping were not totally counteractive. Nitrogen addition and clipping are both associated with increased CWM_SLA_ thereby negatively affected Shannon diversity, reflecting the increased performance of dominant species under nitrogen addition and clipping conditions (Xu et al., 2018). Besides, clipping could also cause niche differentiation among co-occurring species by altering the spacing of functional traits. CV_NND_height_ increased under high clipping intensity, indicating that overgrazing was related to strong selection effect, and the plant height distribution would be limited to a certain range of grazing tolerance. The decrease in CV_NND_SLA_ under moderate clipping intensity indicated that moderate disturbance facilitated the functional differentiation of SLA, probably due to the exclusion of species with similar traits or the colonization of species with distinct traits (Xu et al., 2018). However, we can only observe a significant positive correlation between species diversity and CV_NND_SLA_, suggesting that the net effect of clipping on diversity through niche differentiation was negative. Therefore, these results showed that clipping can mitigate N-induced diversity loss mainly through suppressing dominant species.

## Conclusion

In conclusion, our results show that both the competitive ability of dominant species and niche differentiation (represented by the CWM and CV_NND of different functional traits, respectively) modulated the effects of nitrogen addition and clipping on plant community diversity. Diversity loss due to nitrogen addition were mainly driven by the enhancement of dominant species through increasing CWM. In contrast, clipping influenced species diversity positively by decreasing CWM_height_, and also negatively by increasing CWM_SLA_ and decreasing CV_NND_SLA_, resulting in a weak clipping effect in our experiment. However, the negative effects of nitrogen addition on plant diversity can be alleviated mainly through their opposite effects on CWM_height_. Since nitrogen addition and clipping are two main anthropogenic factors in driving grassland diversity, management strategies could consider incorporating clipping into conservation programs to maintain the diversity of grassland ecosystems.

## Data Availability Statement

The raw data supporting the conclusions of this article will be made available by the authors, without undue reservation.

## Author Contributions

WW and XZ conceived and performed the research. WW and ZR mainly contributed to the investigation and data curation. XW analyzed the data. GD, XZ, and XW made suggestions for the revision of the manuscript. WW wrote the manuscript. All authors have read and agreed to the draft manuscript.

## Conflict of Interest

The authors declare that the research was conducted in the absence of any commercial or financial relationships that could be construed as a potential conflict of interest.

## Publisher’s Note

All claims expressed in this article are solely those of the authors and do not necessarily represent those of their affiliated organizations, or those of the publisher, the editors and the reviewers. Any product that may be evaluated in this article, or claim that may be made by its manufacturer, is not guaranteed or endorsed by the publisher.
